# ZyFISH: A Simple, Rapid and Reliable Zygosity Assay for Transgenic Mice

**DOI:** 10.1371/journal.pone.0037881

**Published:** 2012-05-29

**Authors:** Donal McHugh, Tracy O’Connor, Juliane Bremer, Adriano Aguzzi

**Affiliations:** Institute of Neuropathology, Department of Pathology, University Hospital of Zurich, Schmelzbergstrasse, Switzerland; Louisiana State University Health Sciences Center, United States of America

## Abstract

Microinjection of DNA constructs into fertilized mouse oocytes typically results in random transgene integration at a single genomic locus. The resulting transgenic founders can be used to establish hemizygous transgenic mouse lines. However, practical and experimental reasons often require that such lines be bred to homozygosity. Transgene zygosity can be determined by progeny testing assays which are expensive and time-consuming, by quantitative Southern blotting which is labor-intensive, or by quantitative PCR (qPCR) which requires transgene-specific design. Here, we describe a zygosity assessment procedure based on fluorescent *in situ* hybridization (zyFISH). The zyFISH protocol entails the detection of transgenic loci by FISH and the concomitant assignment of homozygosity using a concise and unbiased scoring system. The method requires small volumes of blood, is scalable to at least 40 determinations per assay, and produces results entirely consistent with the progeny testing assay. This combination of reliability, simplicity and cost-effectiveness makes zyFISH a method of choice for transgenic mouse zygosity determinations.

## Introduction

Transgenic mice are invaluable for studying gene function and modeling human disease. Founder transgenic animals created by pronuclear microinjection typically carry a concatemer of a given transgene at a single, random genomic integration site (“hemizygous”, tg/0). Transgene concatemers are inherited as Mendelian traits, and subsequent breeding of two tg/0 animals from the same founder line can produce offspring that carry two transgenic alleles (“homozygous”, tg/tg).

In many situations, it is useful to maintain a transgenic colony in a homozygous state. Crossings between homozygous mice give rise to 100% transgene-positive offspring, eliminating the need for genotyping and thereby reducing lab costs (e.g. reagents for PCR, labor for cutting tails, and animal caretaking expenses). This is particularly important for experiments requiring multiple transgenic loci [1]. For example, if coexistence of three loci is required, the expected yield of triple transgenic mice from a hemizygous cross will be 12.5%, meaning that the large majority of the offspring from each cross will need to be weaned, genotyped, and eventually eliminated. In such situations, the use of homozygous mice ensures that animals with the desired genotype can be rapidly attained while preventing the generation of animals with unproductive genotypes.

Frequently, homozygosity is desired to achieve higher transgenic protein expression *in vivo.* This may make the function of a protein more obvious, or in cases where a transgene is used to model a disease, homozygosity may lead to an earlier onset or enhancement of clinical phenotypes [2]. Furthermore, the genetic background can have a major influence on transgenic phenotype [3], [4], [5]. A rapid and reliable means of zygosity testing would allow a single homozygous transgenic line to be backcrossed onto multiple genetic backgrounds with relative ease. Finally, experimental constraints may require early discrimination of homozygous and hemizygous mice, e.g. when tissues must be isolated from newborn animals. To date, a number of approaches have been used to test transgene zygosity, each with specific advantages and drawbacks.

### Overview of Zygosity Testing Assays

#### Progeny Testing

The progeny testing method of zygosity determination is based on the principle that homozygous mice, when crossed to non-transgenic mice, will exclusively give rise to mice carrying the transgene. The assay requires the mating of putative homozygous mice with non-transgenic partners (tg/tg×0/0) and subsequent PCR analysis of the F1 offspring for the presence of the transgene. If the transgene is inherited as a Mendelian trait, 100% of the offspring from a tg/tg×0/0 test cross will be tg/0 and will test positive for the transgene in a conventional PCR genotyping assay. In contrast, a tg/0×0/0 test cross will produce a combination of tg/0 and 0/0 offspring with a respective probability of 0.5 for each offspring. This means that the probability of obtaining solely tg/0 offspring from a tg/0×0/0 cross is 0.5*^n^*, where *n* is the total number of offspring which all carry the transgene. Therefore, at least 10 F1 offspring must all test positive for presence of the transgene in order to be reasonably confident (p<0.001, 0.5^10^ = 0.000977) that the animal in question is indeed tg/tg. Although progeny testing is regarded as the gold standard for reliably establishing homozygous transgenic lines, it is a costly and time-consuming endeavor. The 25% probability of obtaining tg/tg offspring from a tg/0 × tg/0 cross means that the progeny of numerous candidates must be screened in order to identify the tg/tg males and females needed to establish a homozygous line. Moreover, some transgenic lines have a reduced reproductive capacity, and by the time sufficient numbers of F1 test progeny have been analyzed to identify both homozygous males and females, subsequent breeding may no longer be possible.

#### Quantitative PCR

Real time or quantitative PCR (qPCR) amplification of genomic DNA has also been used to determine zygosity. This method is much more rapid than the traditional progeny testing, as the DNA of the original tg/0×tg/0 offspring can be directly used to identify tg/tg progeny. This assay would also be expected to be cheaper than progeny testing. However, qPCR does require other time-consuming steps, such as preparation of high-quality genomic DNA and designing highly efficient, transgene-specific primers [6].

The main disadvantage of the qPCR method is that it relies on a DNA amplification technique, which is sensitive to technical errors. Therefore, the reliability of zygosity determinations by qPCR can be obfuscated by the technical imprecision of the assay, which may stem from non-specific amplification, primer dimers being read as signals, or pipetting errors. Although this method has been used successfully to determine transgene zygosity [6], [7], it can also produce ambiguous results that cannot be relied upon [8]. In practice, this method often requires time-consuming optimization for each transgenic line before producing reliable results.

#### Southern blotting

Southern blotting is also frequently used for zygosity testing, and may be one of the more reliable alternatives to progeny testing [7]. Southern blotting offers a number of advantages over other techniques, including the ability to determine various properties of transgene integration, such as approximate copy number per haploid genome, discrimination between individual transgenic lines obtained with the same construct and assessment of orientation of tandem repeats [7]. The fact that Southern blotting does not rely on amplification of DNA means that it does not suffer from variability commonly associated with qPCR. However, Southern blotting is considerably more time-consuming and laborious than PCR analysis, thus making it unsuitable for high throughput assays. In addition, this method raises safety issues, as radioactive probes are routinely used. Furthermore, the amount of genomic DNA needed for the assay requires sizable tail biopsies, which are strongly discouraged by animal welfare committees and should be avoided if possible.

#### Fluorescence *in situ* Hybridization (FISH)

Based on the need for a rapid and reliable zygosity test, and considering the limitations of other frequently used methods, we explored the potential of fluorescence *in situ* hybridization (FISH) as a zygosity test. FISH involves the hybridization of hapten or fluorochrome-conjugated DNA probes to fixed nuclei on slides and subsequent analysis of hybridization loci with fluorescent microscopy. FISH does not suffer from many of the drawbacks associated with other zygosity assays. It is considerably faster than the progeny testing assay. It doesn’t require collection of large biopsies and subsequent isolation of high quality genomic DNA required for Southern blotting. Finally, it does not require primer design or assay optimization as in qPCR, since the plasmid containing the transgene can directly be used to create the probe. Most importantly, the readout of FISH is (1) qualitative, since transgenic alleles are spatially visualized within the cell nucleus and (2) highly redundant, since hundreds of nuclei can be scored from each mouse. Because of these features, we speculated that FISH might be a viable alternative to other commonly used zygosity assays.

Due to lengthy, intimidating protocols, FISH has largely been neglected as a zygosity assay in recent years in favor of qPCR [9]. Here, we have attempted to streamline the FISH zygosity assay by eliminating certain steps or procedures that we suspected might be unnecessary.

Specifically, rather than culturing fibroblasts for slide preparation [10], we used mouse whole blood as proposed by Dinchuk et al. [11], simplified by Paris et al. [12], and further modified by omitting certain slide preparation procedures which proved unnecessary for zygosity testing. This method is a time-saving alternative to fibroblast culturing, more animal-friendly than touch preparations [13] and provides clear hybridization results with either fluorescent or biotin-labeled probes. As the assay only requires a small blood sample, offspring can be tested at a young age. Furthermore, we have developed an unbiased scoring method to standardize the evaluation procedure for mouse zygosity assessment using FISH. We found that our methodology, which we term zyFISH, couples an acceptable level of simplicity with high reliability and at a low overall cost when compared to progeny testing.

## Materials and Methods

A detailed step by step supplementary protocol for the entire zyFISH procedure can be found in the online supplementary material (Text S1, Figure S1, S2, S3, and Video S1). The bench top protocol (Text S1) also contains detailed information on all the reagents used to perform zyFISH including catalog numbers, preparation and storage of stock solutions. We also provide timing (Table 1) and troubleshooting advice (Table S1).

**Table 1:** Timing of a typical zyFISH assay.

Step*	Procedure	Timing
1–8	Probe construction	2 h
9		Overnight
10–14		1 h
15–30, 16B	Bleeding and slide preparation	2–3 h for 20 mice including 1 h incubation step
31–38	Hybridization	45 min
39		generally performed overnight
40–44, 41A	Washing and counter-staining	1 ½ h
40–44, 41B		4 h
45–49	Scoring and evaluation	2–10 min per slide, depending on nuclei density on slide and microscopy experience of the investigator

* Refers to steps in the supplemental zyFISH protocol (Text S1).

### Animals and Ethics Statement

*Tg*a20 transgenic mice were generated *via* pronuclear injection of a “half-genomic” *Prnp*-encoding construct from which the large intron (intron 2) was deleted, containing 5.5 kb of 5′- and 2 kb of 3′-flanking sequences [2] into the oocytes of *Prnp^O/O^* mice [14]. *Tg*a20 mice were created and maintained on a mixed (C57BL/6×129/Sv) background and transgenic carriers were identified by PCR with the primers 5′-CCTGGGACTCCTTCTGGTACCGGGTGACGC-3′ and 5′-CAACCGAGCTGAAGCATTCTGCCT-3′ as previously described [2]. C57BL/6 mice were purchased from Harlan laboratories and then bred and maintained in-house. *Tg*a20 mice were backcrossed to C57BL/6 mice and offspring were selected for further breeding that harbored the wild-type *Prnp* gene (5′-ATACTGGGCACTGATACCTTGTTCCTCAT-3′ and 5′-GCTGGGCTTGTTCCACTGATTATGGGTAC-3′) and the *tg*a20 transgene. *Tg*a20 mice were backcrossed in this manner for over 12 generations to yield *tg*a20 mice on a predominantly C57BL/6 background that no longer harbored the *Prnp^O/O^* neo cassette (C57BL/6-*tg*a20). C57BL/6-*tga*20 mice were then used for zyFISH analysis shortly after weaning. All experiments using wild-type or transgenic mice conform to the rules and regulations for the Protection of Animal Rights (Tierschutzverordnung) of the Swiss Bundesamt für Veterinärwesen and have been approved by the Animal Welfare Committee of the Canton of Zürich; permit number 200/2007.

### Probe Preparation for FISH

Biotin-labeled probes were prepared with the Biotin-nick translation mix for *in situ* probes (Roche Applied Science) according to the manufacturer’s instructions and published [15], [16] procedures with our own modifications. The *Prnp*- “half-genomic” plasmid (pPRPHG [2]) was diluted to 62.5–100 ng/µl in a total volume of 80 µl and added to 20 µl of the Biotin-nick translation enzyme mixture. Fluorochrome-labeled probes were prepared with the Nick translation kit for *in situ* probes (Roche Applied Science) according to the manufacturer’s instructions and published [15], [16] procedures with our own modifications. The *Prnp-* “half-genomic” plasmid [2] was diluted to 62.5–100 ng/µl in a total volume of 77 µl. This was added to 3 µl of the dNTP mixture (including fluorescein-dUTP, Roche Applied Science, cat. no. 11 636 154 910) and 20 µl of the nick translation enzyme mixture. Reaction mixtures were incubated at 20°C for 90–110 min to achieve probe sizes with a length of 100 to 1,000 bp (Figure S1). The labeled DNA was washed with the GE Illustra purification kit according to the recommendations of the manufacturer. Probe concentration was measured by UV spectrometry. 20 µg of unlabeled salmon sperm DNA (Invitrogen) and 2–3 volumes of 100% (v/v) ethanol were added to the labeled DNA and precipitated overnight at −20°C. The precipitate was centrifuged at 16,000×g for 20 min at 4°C. The supernatant was then discarded, and the remaining pellet was dried with a vacuum centrifuge. The pellet was resuspended in 100% (v/v) deionized formamide to a concentration of 10–20 ng/µl probe DNA. The DNA was denatured for 5 min at 72°C then place on ice. One volume of hybridization solution containing 4× SSC, 0.2% (w/v) nuclease-free BSA, and 20% (w/v) dextran sulfate was added to the denatured probe. This probe mix was either stored at −20°C or used directly for hybridization. Mouse major satellite (MMS) probes were created with PCR and prepared for hybridization as previously described [15].

### Slide Preparation

Mouse whole blood (30–100 µl) was obtained from the lateral tail vein by making a small incision with a sterile scalpel (see supplementary video S1), dropped directly into 10 ml of 65 mM KCl, and held at room temperature (RT) for 1 h as previously described [12]. The suspension was centrifuged at 400×g for 5 min at 4°C and all but 500 µl of the supernatant was discarded. The pellet was resuspended in freshly prepared fixative (25% (v/v) acetic acid and 75% (v/v) methanol, 500 µl) and then centrifuged for 2 min at 1500×g at 4°C. The nuclei suspension was washed an additional two times with 1 ml of fresh fixative. After the final centrifugation, all but 50–100 µl of the supernatant was removed. The nuclei suspension (50 µl) was either dropped onto microscope slides and stored at RT in a closed slide box for hybridization the following day or stored for 11 months at −20°C with an additional milliliter of fixative. Upon storage, nuclei were washed once and applied to slides.

### Hybridization and Detection

The hybridization and detection procedure was performed essentially as previously described [16] with some minor changes. Slides with interphase lymphocyte nuclei were incubated in 70°C deionized formamide 70% (v/v), 2× SSC at pH 7.0 for 2 min, cold 70% (v/v) ethanol followed by room temperature 80%, 95%, and 100% (v/v) ethanol for 2 min each. Slides were air dried, and 20 µl of probe mix was added to the center of each slide, covered with Parafilm, and incubated for 14–18 h in a dark moist chamber at 37°C. Slides were washed for 15 min each in 2× SSC 50% (v/v) formamide at 39°C, 2× SSC at 39°C, and finally in 1× SSC at RT followed by incubation in 4× SSC at RT for 5 min. At this point, slides hybridized with fluorescently labeled probes were counterstained with 50 ng/ml Hoechst-33342 (Invitrogen) in 4× SSC at RT for 5–10 min, and then rinsed in 1× SSC. Coverslips were mounted with fluorescent mounting medium (Dako). Samples hybridized with biotin-labeled probes were blocked in 1% (w/v) BSA in 4× SSC for 30 min at 37°C. Then 50 µl of 10 ng/µl Alexafluor 488-conjugated streptavidin (SA-488, Invitrogen) in 1% (w/v) BSA, 4× SSC was applied to each slide, covered with parafilm strips and incubated for 1 h in the dark moist chamber at 37°C. Slides were then agitated sequentially in 0.1% (v/v) Triton X-100, 4× SSC and then twice in 4× SSC for 10–15 min each at RT. Counter-staining was performed the same as for direct fluorescently probed slides.

### Images, Scoring and Statistical Evaluation

Slides were analyzed by fluorescent microscopy with an Olympus BX61 TRF equipped with a CCD camera (Olympus, F-View Soft Imaging system) and a cold light source (EXFO_XI120PC-xl supplied by Olympus). Images were acquired with Analysis 5.0 image analysis software (Olympus).

To be eligible for scoring, nuclei had to be intact and display clear edges. Further, they were required to neither overlap nor touch other nuclei, as visualized by the nuclear dye in the Blue/Cyan filter. Hybridization signals were required to be uniformly bright among different nuclei and display similar intensity within nuclei containing more than one signal. Nuclei with auto-fluorescent debris in the vicinity were excluded from the scoring procedure. Upon completion of scoring a set target (e.g. 50 nuclei) the remaining nuclei within the field of view were scored.

All scored nuclei from each slide were categorized into one of two groups, which we termed POS and NEG, depending on the number of signals they displayed. Category POS contained all nuclei determined to have two hybridization signals. Category NEG contained all remaining nuclei (i.e. containing 0, 1, 3 or more signals). Animals were assigned the zygosity status “homozygous” if they showed a significantly different distribution of the categorical data (POS and NEG) from a known hemizygous control animal as calculated with a two-tailed Fisher’s exact test with a significance level α = 0.001. Remaining animals were assigned the zygosity status “hemizygous” if they were positive for the transgene by conventional PCR genotyping.

### Statistical Analysis

Categorical data was assessed with a two-tailed Fisher’s exact test with a significance level α = 0.001. The power of a two-tailed Fisher’s exact test of equal proportions with a significance level α = 0.001 for different sample sizes was calculated with nQuery Advisor® 6.0 software. Comparison of mean values of scoring results was performed with a two-tailed unpaired t-test with a significance level α = 0.0001.

## Results

### ZyFISH is a Rapid and Reliable Protocol for Zygosity Determination

**Figure 1:**
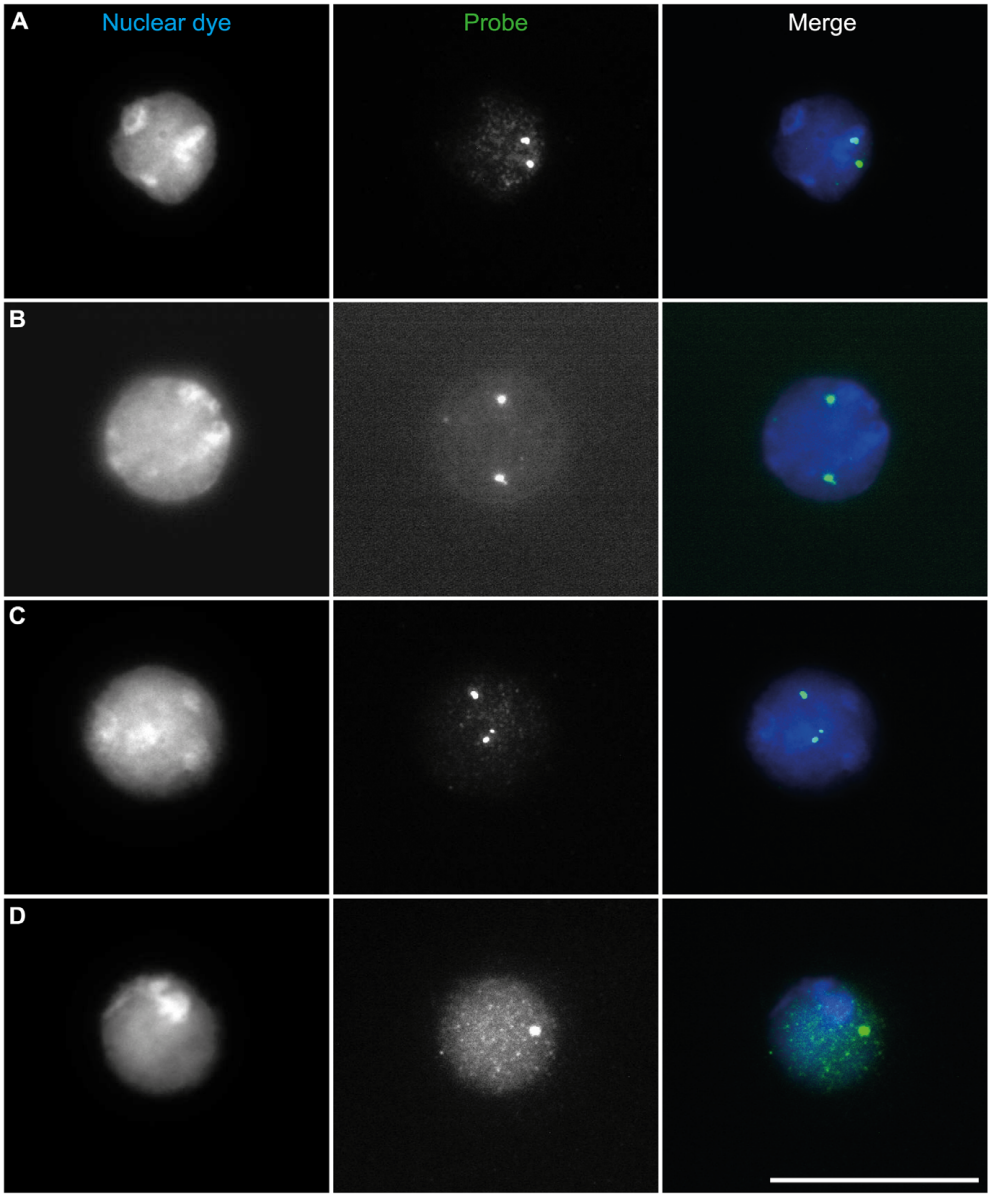
Categorization of transgenic nuclei using the zyFISH scoring system. Lymphocyte nuclei were isolated from C57BL/6-*tg*a20 transgene-positive mice, fixed, and mounted on slides as described. FISH was performed with a fluorescein-labeled probe (Probe) and counter-stained with Hoechst-33342 (Nuclear dye). Scale bar = 20 µm. (A), (B) Examples of nuclei which show two bright fluorescent signals and were categorized as positive (or POS). (C) Three distinct hybridization signals were categorized as negative (or NEG). (D) One distinct hybridization signal, in this case over a diffuse intranuclear background signal, was also categorized as negative.

**Figure 2:**
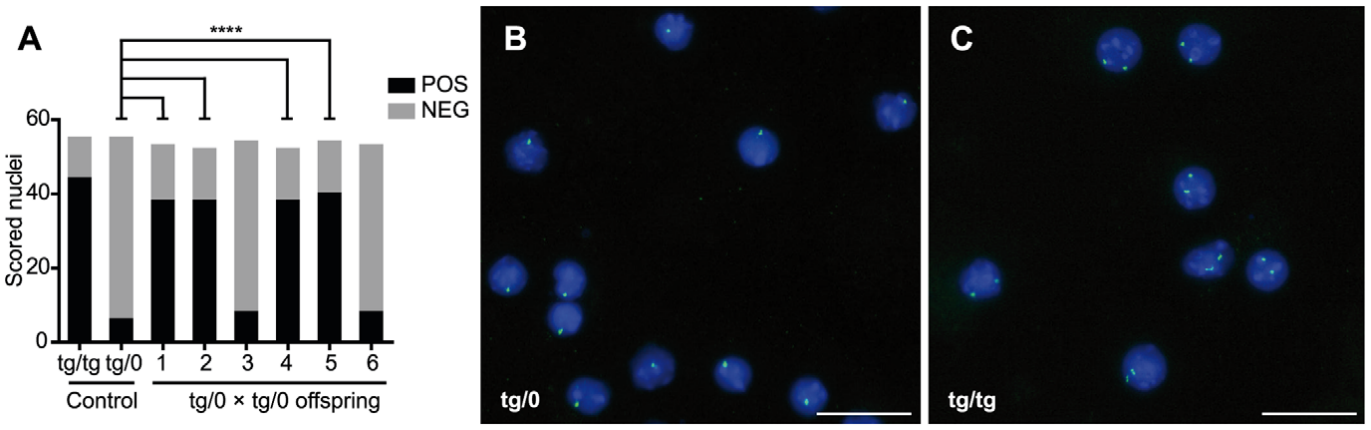
Determination of zygosity using zyFISH. (A) Results of zyFISH performed as described on fixed lymphocyte nuclei from six C57BL/6-*tg*a20 transgene-positive offspring of unknown zygosity from a *tg/0*×*tg/0* C57BL/6-*tg*a20 cross. A minimum of 55 nuclei were scored per mouse and categorized either as positively-scoring (POS) or as negatively-scoring (NEG) according to the rules in step 46 of the supplementary zyFISH protocol (Text S1) and Figure 1. Total numbers of scored lymphocytes (both POS and NEG) are shown for each offspring in comparison to homozygous (tg/tg) and hemizygous (tg/0) controls. P-values (p) were calculated with a two-tailed Fisher’s exact test for each mouse in comparison to the hemizygous control (tg/0), **** = p<0.0001. Mice 1, 2, 4 and 5, all had significantly different counts of POS- and NEG-scored nuclei versus the hemizygous control in the Fisher’s exact test and were therefore regarded as homozygous. Mice 3 and 6 do not have a different proportion of nuclei categories and were thus regarded as hemizygous by default, as all mice tested positive for the transgene in conventional PCR genotyping. (B,C) Representative merged images from microscopy view fields of zyFISH are depicted. Lymphocyte nuclei were isolated from hemizygous (tg/0; B) and homozygous (tg/tg; C) NGPI155 transgenic mice, fixed and mounted on slides as described. FISH was performed with a fluorescein-labeled probe and counter-stained with Hoechst-33342. Scale bar = 20 µm.

To test the zyFISH protocol, we employed *tg*a20 (C57BL/6-*tg*a20*^tg/0^*) transgenic mice overexpressing the cellular prion protein (PrP^C^) on a predominantly C57BL/6 background [2]. In order to obtain PrP^C^-overexpressing mice homozygous for the *tg*a20 transgene (C57BL/6-*tg*a20*^tg/tg^*), we crossed two C57BL/6-*tg*a20*^tg/0^* mice. We analyzed the first litter of seven pups from this cross *via* conventional PCR to identify transgene-positive animals. Only one of the offspring was transgene-negative and was eliminated from further analysis. To distinguish C57BL/6-*tg*a20*^tg/tg^* from C57BL/6-*tg*a20*^tg/0^* animals in this litter, blood was collected from the remaining six mice (Video S1) and examined by zyFISH, as described in the Materials and Methods section and the detailed supplementary benchtop protocol provided (Text S1). Briefly, probe DNA was constructed directly from the transgene plasmid by labeling with dUTP-fluorescein *via* nick translation. Whole blood preparations from mice were fixed, mounted on glass microscope slides, and hybridized with labeled probe. Blood preparations from known C57BL/6-*tg*a20*^tg/0^* and C57BL/6-*tg*a20*^tg/tg^* mice were used as controls. Using the specific criteria outlined in our scoring method (see Materials and Methods for detailed scoring procedure), nuclei were classified as either positive (POS) if they contained two clear hybridization loci (Figure 1 A & B) or negative (NEG) if they contained zero, one, three, or more hybridization signals (Figure 1 C & D). Just over 55 nuclei were scored per mouse, resulting in the identification of four homozygous and two hemizygous mice (Figure 2A). To validate our results against the progeny assay, all six mice were then crossed to non-transgenic animals and their offspring were genotyped by PCR. Our zygosity predictions were confirmed in all tested mice by the progeny assay (Table 2), thus confirming zyFISH as a reliable and fast (3 days from beginning to end; Table 1) method of zygosity testing. To date, we have applied zyFISH successfully to more than 250 samples from 7 different transgenic lines. Direct prospective comparison with the progeny assay as described (Figure 2 A, Table 2) was performed in 3 lines (*tg*a20 and 2 novel unpublished lines: NGPI155 and NGPI177) with a total of 20 mice and resulted in the confirmation of zygosity status for each mouse predicted by zyFISH. Analysis of offspring sired by zyFISH-tested transgenic mice, with PCR and/or zyFISH has thus far yielded no unexpected results. Importantly, zyFISH is based on a qualitative readout (Figure 2B & C) for each mouse, which is highly reliable and eliminates the need for zygosity confirmation using the progeny assay.

**Table 2:** Comparison of ZyFISH and progeny testing assay for zygosity determination in C57BL/6-tga20 littermates.

Mouse ID	ZyFISHResult	PCR positive F1offspring (tg+/n)	P/(tg/0)
1	tg/tg	100% (15/15)	3.1E-05
2	tg/tg	100% (21/21)	4.8E-07
4	tg/tg	100% (15/15)	3.1E-05
5	tg/tg	100% (20/20)	9.5E-07
3	tg/0	44% (4/9)	N/A
6	tg/0	63% (5/8)	N/A

Six C57BL/6-*tg*a20 transgene-positive mice with predicted zygosity (Figure 2A) according to zyFISH (ZyFISH Result) were each crossed with a C57BL/6 non-transgenic mate. The resulting offspring were genotyped *via* conventional PCR (tg+  =  number of transgene-positive offspring, n  =  total number of offspring). All mice predicted by zyFISH to be tg/tg had 100% transmittance of the transgene to their offspring, whereas predicted hemizygous mice transmitted the transgene with at a rate of ∼50% (PCR positive F1 offspring). For each of the tg/tg mice we calculated the probability of a tg/0 mouse to sire this many tg+ offspring in succession, by 0.5^n^ (P/(tg/0)). Not applicable (N/A).

### ZyFISH has a Lower Threshold between 36 kb and 60 kb of Transgenic DNA per Haploid Genome

In FISH, the intensity of a hybridization signal depends partly on the length of the DNA sequence detected within the nuclei. *Tg*a20 mice carry about 30 copies of a 12 kb transgenic construct per haploid genome [2] and transgene hybridization signals were readily detected with the zyFISH protocol. To determine the lower threshold of transgenic signal detection for the zyFISH protocol, we probed nuclei from transgenic mice with lower copy numbers each with a construct length of 12 kb. Using the zyFISH protocol with biotin-labeled probes detected with SA-488, we could reliably detect transgenic loci from mice with five transgene copies (PrP_ΔCDs_ mice, line Tg42 [17]). Clear and distinct hybridization signals were readily viewed with a wide-field fluorescent microscope at a 60× magnification. However, we were not able to detect the transgenic loci from mice with three transgene copies per haploid genome (PrP_ΔCD_ mice, line Tg1047 [18]). Therefore, we estimate the lower sensitivity threshold of zyFISH to be between ∼36–60 kb of transgenic DNA per haploid genome. An overview of transgenic lines tested with zyFISH can be viewed in the supplementary material (Table S2).

### The zyFISH Scoring Method Produces Similar Results among Different Investigators

**Figure 3:**
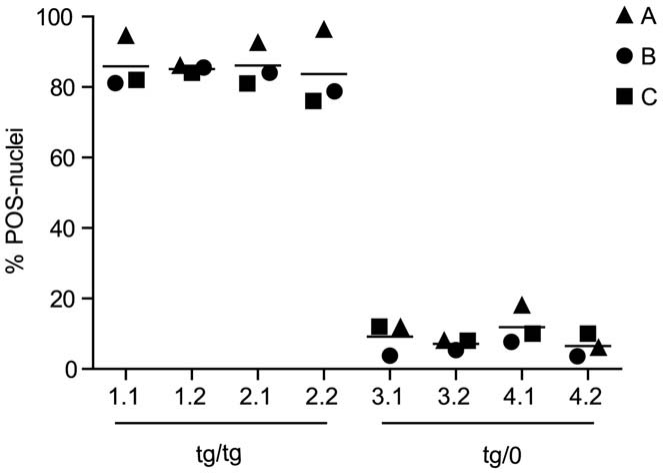
Zygosity assignment by different investigators using zyFISH. Analysis of samples obtained from four C57/Bl6-*tg*a20 transgenic mice with known zygosity (1–4); two slides were produced from each sample (e.g. 1.1 and 1.2). Slides were hybridized with a biotin-labeled probe, detected with SA-488 as described and then keyed by a third party. These eight keyed slides were scored by three investigators (A, B and C) independently of each other. A target of 50 nuclei was set to be scored according to the rules described in step 46 of the supplementary zyFISH protocol (Text S1). A mean number of 52 nuclei were scored. All investigators scored nuclei similarly and in accordance with the true zygosity of each mouse from which the slide was produced (mean 85.31% POS-nuclei +/−6.243% SD for tg/tg-derived samples, mean 8.70% POS-nuclei +/−4.050% SD for tg/0-derived samples).

In order to assess whether variations in slide preparations or inter-observer variability would impact zygosity determinations by zyFISH, we created two slides each from four mice of known zygosity, as determined by the progeny assay. After hybridization and detection of the biotinylated probe *via* SA-488, slides were keyed by an independent third party. Three blinded observers then scored a target number of 50 nuclei according to the rules detailed in the scoring procedure (step 46 of the supplementary protocol; Text S1) independently of each other. True zygosity of each mouse was revealed only after all observers had completed scoring. Observer C had extensive experience with the protocol prior to this experiment, observer A had intermediate experience (had performed it four times before) in scoring nuclei, whereas observer B had no prior experience in using zyFISH. All three independent observers could correctly determine the zygosity of each source mouse on each slide (Figure 3), indicating that the rules, as detailed in step 46 of the supplementary protocol (Text S1), were indeed sufficient to ensure homogeneous and accurate scoring results. Moreover, independent slide preparations from the same mouse yielded the same zygosity results.

### Scoring Thirty Nuclei is Sufficient for Accurate Zygosity Assignment

**Figure 4:**
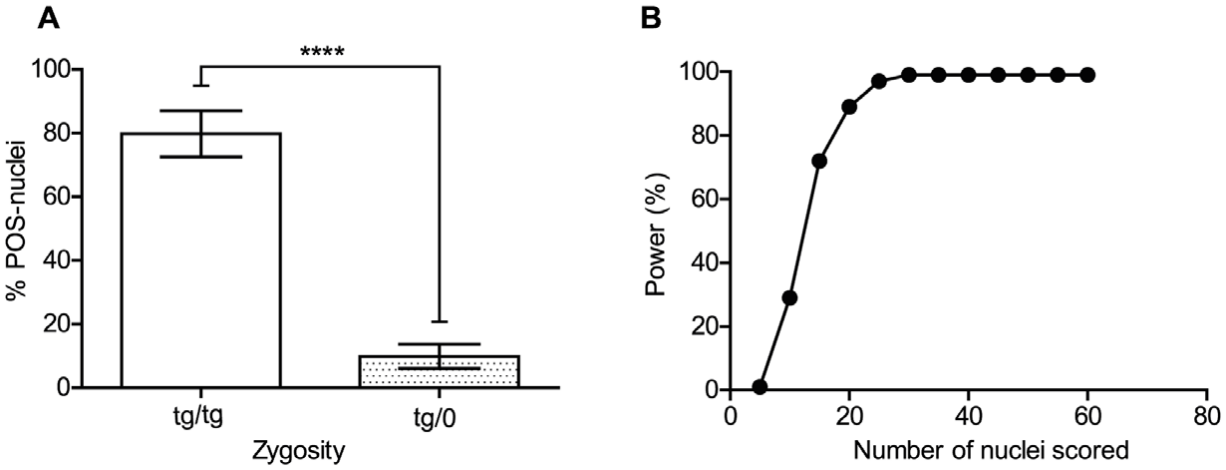
Accurate zygosity assessment scoring thirty nuclei by zyFISH. (**A**) Cumulative data from four independent experiments illustrating the mean percentage of positively-scored nuclei (% POS-nuclei) in lymphocytes from C57BL/6-*tg*a20 mice with known zygosity. Homozygous animals had a mean of 79.78% (SEM +/−2.414, n = 9 mice) POS-nuclei, whereas tg/0 animals had a mean of only 9.875% (SEM +/−1.342, n = 8 mice) POS-nuclei. Comparison of the mean values from each group was performed with a two-tailed unpaired t-test, **** = p<0.0001. Error bars display standard error of the mean (SEM). (**B**) Determination of the minimal sample size needed for zyFISH assay. The power of a two-tailed Fisher’s exact test with the mean category proportions of tg/tg and tg/0 mice (Figure 2E) and a significance level α = 0.001 was calculated with nQuery Advisor 6.0 for increasing numbers of scored nuclei. Power is plotted against the total numbers of nuclei scored per slide. As the sample size surpasses 30 nuclei for both the tested mouse and the hemizygous control, the power of Fisher’s exact test reaches 99%. Scoring a minimum of 30 nuclei is sufficient for reliable zygosity determination of C57/Bl6-*tg*a20 mice using zyFISH.

Previously published FISH-based methods recommended scoring between 50 and 100 nuclei or metaphase spreads [10], [11], [13], [19]. But with the necessity of employing such methods on a large scale and possibly on a bi-weekly basis, we wanted to assess the minimal sample size needed for correct zygosity determination using our scoring system. The calculation of the minimal sample size was based on the mean fractions of nuclei categories (POS and NEG) from mice with known zygosity (Figure 4A) from four different experiments (9 mice for C57BL/6-*tg*a20*^tg/tg^* and 8 mice for C57BL/6-*tg*a20*^tg/0^*). These mean fractions were used to calculate the power of a two-tailed Fisher’s exact test of equal proportions with a significance level α = 0.001 for different sample sizes (n  =  number of nuclei scored from the tg/0 control slide and from the slide of the putative tg/tg mouse). We used nQuery Advisor® 6.0 software to perform these calculations. We determined that under these conditions, with a sample size of 30 nuclei, the Fisher’s exact test already reached a power of 99% (Figure 4B). Consequently, we were able to further abbreviate the readout of zyFISH and discriminate between homozygous and hemizygous *tg*a20 mice with no reduction in accuracy.

### Long-term Storage of Nuclei does Not Affect Reliability of zyFISH

**Figure 5:**
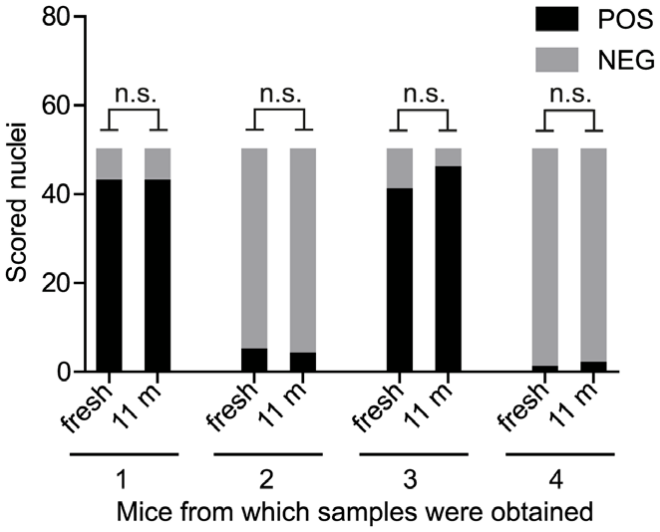
Accurate zygosity assessment by zyFISH after prolonged storage of nuclei. Whole blood from four transgene-positive tg*a20* mice (1–4) was collected and the lymphocyte nuclei were isolated and fixed as described. One half of each of the lymphocyte nuclei suspensions was immediately used for zyFISH, while the other half was stored with an additional milliliter of fixative at −20°C. After 11 months of storage, the stored nuclei were washed and applied to slides. ZyFISH was performed under identical conditions with a fluorescein-labeled probe. Scoring of 50 nuclei per slide revealed no significant differences (two-tailed Fisher’s exact test, α = 0.05) between fresh samples (fresh) and those that underwent long-term storage (11 m).

In order to assess whether long-term storage of fixed nuclei would affect the outcome of zyFISH scoring results, we compared fresh and stored samples from four transgene-positive *tg*a20 mice (Figure 5). Mice 1 and 2 were offspring of unknown zygosity from a tg/0×tg/0 C57/Bl6-tga20 cross, mouse 3 had previously been determined to be homozygous by progeny testing, and a sample from mouse 4 (*tg*a20*^tg/0^* on a *Prnp* ^O/O^ C57BL/6×129/Sv mixed background) served as a hemizygous control. One half of each of the lymphocyte nuclei suspensions was immediately used to prepare slides and tested the following day, while the other half was stored for 11 months in 1 ml of fixative solution at −20°C. ZyFISH was performed in both instances with a fluorescein-labeled probe. The morphology seemed unaffected by the storage and, crucially, we did not observe any difference in category distribution between the freshly produced slides and slides produced from the stored samples. The respective zygosity of mouse 1 and 2 as predicted by zyFISH was confirmed by progeny testing (data not shown).

### ZyFISH is More Cost-effective than the Progeny Testing Assay

Cost control is crucial to efficient basic research, especially when it comes to day-to-day determinations or measurements. Although zyFISH was considerably faster than the progeny testing assay, cost could still be the deciding factor for other research laboratories working with transgenic animals, since we obtained equivalent results with progeny testing and zyFISH (Figure 2A and Table 2). The difference in expenditure between assays was estimated by comparison of the *additional* costs incurred by both approaches per mouse tested (Table 3). We defined additional costs as new expenses any standard molecular biology laboratory that works with transgenic mice would incur per mouse by using either method after the cross of two tg/0 mice. In zyFISH, reagents and enzymes made up the main part of the costs, whereas in the progeny testing assay, animal housing [20] contributed to the majority of the expenditure. To put this data in context of additional labor needed to perform each assay, we estimated the time a trained technical assistant would need to complete the respective assays and included the payroll in the total costs, based on an eight-hour workday with a salary of 90′000 CHF a year.

**Table 3:** Cost comparison of the progeny testing assay with zyFISH.

PTA (n = 6)	Cost (CHF)	ZyFISH (n = 6+1 control)	Cost (CHF)
Housing cost of 1 tg/? litter post-weaning, pre-breeding	56	Cost of probe	24.7
Cost of mice post-weaning pre-breeding	72	Cost of hybridization	4.5
Housing cost for the duration of breeding	214.8	Cost of detection and scoring	15.1
Cost of mice for the duration of breeding	107.4	Miscellaneous (tubes, slides, tips, general supplies, not included above)	5
Cost of PCR genotyping offspring	26.4	Payroll technician for entire 10 h zyFISH protocol	468.8
Payroll technician for entire PA	750		
Total cost for 6 mice	1226.6	Total cost for 6 mice	518
Total cost per mouse	204.4	Total cost per mouse	86.3

“Additional costs” (defined as costs incurred after breeding two *tg/0* mice, which are the same for both assays) associated with the progeny testing assay (PTA, Table 2) for 6 transgene-positive mice of unknown zygosity (tg/?) are compared with additional costs associated with zyFISH (ZyFISH, Figure 2A). “Cost of mice” and “Cost of Housing” are based on the rates for renting cage space per mouse and cage at our animal facility [20]. We incurred 40 days of pre-breeding costs, including housing of putative homozygous littermates post-weaning and cost of prospective mates (6) and mean 60 days of breeding costs, including six breeding cages, each with two mice. “Payroll technician” cost estimations are based on 1 full-time technician paid 90,000 CHF/year working 8 h days. Estimation of cumulative technician time for conducting PCR and running gels for 110 samples in total was 8 h. Estimation of cumulative technician time spent on weaning, biopsies (92 biopsies), mouse health checks and cage changes was 8 h over the 100 days. Cost of “probe,” “hybridization,” and “detection and scoring” includes all reagents, chemicals, enzymes and solutions used for each part of the protocol for seven slides. Cost of “PCR genotyping” includes the cost of Taq polymerase enzyme solution for 110 samples. Costs are reported in Swiss francs (CHF) and are rounded to the first decimal point.

We found that zyFISH not only delivered results more than 3 months earlier, but also at a cost of 86.3 CHF per mouse, compared to 204.4 CHF for the progeny assay. Although there may be local differences regarding the costs of reagents, salary and animal housing, the relative difference in cost between the two assays is likely to be similar in other areas. These considerations speak strongly in favor of zyFISH as a superior zygosity testing option for any laboratory engaged in transgenic research.

## Discussion

Zygosity assessment of transgenic mice is a common task for laboratories engaged in transgenic mouse research. Here, we investigated FISH as a method for determining transgene zygosity status based on the evaluation of hybridization signals. The use of FISH as a zygosity assay has been described before; however, thorough prospective validation of FISH performance against the “gold standard” progeny test has, to our knowledge, never been reported, and published protocols contained steps or procedures that we suspected might be unnecessary such as fibroblast culturing and slide preparation according to cytogenetic procedures, the use of two separate probes, or the evaluation of 50–100 cells or nuclei. Furthermore, previously published studies were generally performed in transgenic lines with a large amount of transgenic DNA per haploid genome (128 kb–2′600 kb [10], [11], [12], [13], [19]), and the lower threshold of the assay had not been established. Here, we validated zyFISH by direct comparison to progeny testing and have determined the sensitivity of the assay. This protocol has been successfully employed with biotin-labeled probes for plasmid-based transgenic mouse lines with previously reported copy numbers as low as 5 and 6 (PrP_ΔCDs_ mice, lines Tg42 and Tg40 respectively [17]) but not 3 or 2 (PrP_ΔCD_ mice, line Tg1047 [18] and AlbLTαβ mice [21] respectively) (Table S2). Based on these results, we conclude that the lower threshold of detection for this protocol is between 60 kb and 36 kb of transgenic DNA per haploid genome. Amplification techniques, confocal microscopy, or long exposure time during image acquisition may further increase the sensitivity of this assay. However, such techniques are also likely to prolong the protocol.

Zygosity results obtained with zyFISH in different transgenic lines have been entirely consistent with the progeny testing results. Further, a modified version of the protocol has been created for animal housing facilities that are not in the immediate vicinity of the molecular biology lab processing the samples. In addition, we developed a concise, streamlined scoring system for nuclei with simple statistical evaluation which ensures uniform and accurate results. In addition to speed and reliability, a direct comparison of the incurred costs in progeny testing versus zyFISH show that zyFISH is the cheaper option. Finally, the number of offspring generated during the test crosses in the progeny assay for the mere assessment of zygosity status has animal-welfare implications. In many countries, including Switzerland, investigators carry the responsibility to minimize the number of superfluous animals that are ultimately euthanized. Considerations of this nature are mandated by legislation in line with the principals to replace, refine and reduce the use animals in research in some countries (e.g. the Swiss Federal Act on Animal Protection of 16 December 2005, the Swiss Animal Welfare Ordinance of 23. April 2008 and the European Union Directive 2010/63/EU of 22 September 2010).

### Experimental Design Considerations

The standard zyFISH experimental protocol should include control slides from at least one known hemizygous transgenic mouse. The offspring from a breeding between two tg/0 mice can be tested at a young age shortly after conventional PCR genotyping to exclude wild-type offspring from further assessment. Homozygous mice are usually not available when zyFISH is first employed. However, due to the nature of the scoring method, they are not required as a positive control. Upon scoring, the statistical analysis is conducted as a comparison of groups, whereby true homozygous animals will show a significantly different distribution of POS versus NEG hybridization categories compared to a known hemizygous control animal.

It should also be noted that a single probe can hybridize to different transgenic loci if their sequences strongly overlap. However, using a combination of an appropriate breeding strategy, conventional PCR genotyping, and zyFISH, researchers may take advantage of this property of FISH probes to assess double transgenic animals with a single probe. For instance, the probe we used to detect the *tg*a20 transgene [2] was successfully used to detect other *Prnp*-transgenes created with the same vector (*tg*a20 [2]; PrP_ΔCDs_, lines Tg42 and Tg40 [17]; PrP _Δ32–93,_ line C4 [22] and a further 2 unpublished lines).

### Probe Preparation and Use

One major advantage of zyFISH over other zygosity assessment procedures is that the probe generation procedure is uniform for different transgenes, and no transgene-specific design is required. The template DNA used for FISH probe generation should ideally be the same as that of the DNA used for generation of the transgenic mouse line. The prokaryotic plasmid backbone may be excised if desired, but in our experience its presence did not influence the hybridization efficiency or specificity to the extent that it would interfere with the evaluation of zygosity status.

There is a variety of methods to label DNA probes [15]. Here, we describe two labeling methods, which, in our experience, have proven useful for zyFISH: namely, direct fluorescent-labeling and biotin-labeling with nick translation. In our hands, fluorescent-labeled probes produced clearer hybridization signals with less background but required careful handling due to the photolability of the fluorescent conjugates. In contrast, biotin-labeled probes produced slightly more intense signals, and the detection procedure, although slightly longer, was simplified by the fact that photolabile avidin-fluorochrome conjugates are only employed in the later steps of the assay. We recommend the use of the more sensitive biotin-labeled probes for the detection of low copy number transgenes and for transgenics with an unknown copy number. However, if the total length of the hybridization target DNA exceeds 100 kb, both labeling strategies can be used with equal success. If hybridization signals are sufficiently intense, the use of direct fluorochrome labeling might be considered in subsequent experiments. The use of Strepavidin Alexafluor 488 conjugate is not critical. We have also used Avidin D Texas Red conjugate (Vector labs, cat. no. A-2006) with similar results. Fluorochrome conjugates that emit light within the green spectrum seem more user-friendly when scoring nuclei at the microscope, but this depends on the microscope filters sets that are available as well as individual preference.

The extent of probe DNA digestion during Nick translation is absolutely critical. If the probe is too long it will result in high, blotchy background on free surfaces of the slide and true hybridization signals within the nuclei will be less intense due to poor hybridization efficiency [15]. However, extending the reaction time considerably may result in degradation of the probe and a reduced yield of labeled DNA probe (Troubleshooting Table S1). We recommend the use of commercially available kits that have titrated enzymes, which produce probe DNA fragments between 100 and 1,000 bp in length (Figure S1). These are usually specified as enzyme mixtures explicitly for *in situ* probes. We have also used Nick translation mixtures that do not digest the DNA as readily; however, to achieve equal hybridization results we had to resort to subsequent digestion of the labeled DNA with DNAse I.

Hybridization duration can be varied depending on the size of the target sequence (longer incubation times for smaller targets). According to the literature, incubation times between 14–18 h are recommended [16]. In our hands, four hours were completely sufficient in the case of nuclei obtained from *tg*a20 transgenic mice hybridized with biotin-labeled probes, but longer incubation times of 16–19 h did not lead to an increase in unspecific hybridization signals (data not shown).

When employing zyFISH for the first time, it is advisable to use a probe directed against MMS sequences as a control on a separate slide, since MMS are highly repetitive and produce strong hybridization signals (Figure S3). Protocols for the generation of MMS FISH probes with PCR have been described in detail elsewhere [15]. MMS probes will display a hybridization pattern similar to that of the nuclear dye Hoechst-33342, as MMS regions detected with MMS FISH probes are double-stranded DNA with dA- and dT-repeats [23] to which the Hoechst dyes bind preferentially [24]. Finally, when testing new probes for transgenic lines, it is advisable to use non-transgenic mice with the same genetic background as the transgenic mice as a negative control.

In summary, we conclude that zyFISH is a surprisingly straight-forward and convenient method of zygosity assessment for transgenic research. ZyFISH is rapid, reliable, cost-effective, and requires no transgene-specific design or optimization. The protocol we have employed can reliably detect alleles from plasmid-based transgenic mice with five transgenic copies per haploid genome. For laboratories that do not already have a preferred method of zygosity assessment, are experiencing technical difficulties with another zygosity assay, or are currently using the progeny assay, zyFISH may be a viable alternative.

## Supporting Information

Figure S1 **Optimal FISH probe length.** A typical example of a biotin-labeled probe that produced clear signals and low background fluorescence when hybridized to fixed lymphocyte nuclei obtained from C57BL/6-*tg*a20*^tg/0^* mice. Following nick translation, 3 µl of probe was mixed with loading dye and denatured for 3 min at 95°C, placed on ice shortly, and then loaded alongside a 100 bp marker (100 bp plus gene ruler, Fermentas) on a 1% (w/v) agarose gel with 0.2 µg/ml ethidium bromide in TAE. Correct probe smears for FISH range from 100–1000 bp, the majority of the probe running below 500 bp.
(TIF)

Figure S2 **Artifacts that may influence zyFISH scoring.** (A) Merged FISH image of a fixed lymphocyte nucleus from a homozygous C4 transgenic mouse carrying 25 transgene copies and a vector length of 12 kb per haploid genome [22] hybridized with a fluorescein-labeled probe. Occasionally, the hybridization signals appear as a string of hybridization spots (left signal). More often, signals will have an elongated appearance (right signal). Scale bar = 20 µm. (B) Merged FISH image with fluorescent debris. These two nuclei would be excluded from the scoring process as the debris overpowers any specific signals from within the nuclei. Scale bar = 20 µm.
(TIF)

Figure S3 **Example of mouse major satellite FISH images.** FISH was performed as described in the Materials and Methods section on fixed lymphocyte nuclei from a C57BL/6 mouse with a fluorescein-labeled probe (Probe) for mouse major satellite (MMS) sequences [15] and counterstained with Hoechst-33342 (Nuclear dye), scale bar = 20 µm. The nuclear dye signal and the signal obtained with the MMS probe co-localize (Merge) within the same regions of the nucleus. The MMS probe can thus be used as a positive control for the FISH procedure when first using this assay.
(TIF)

Text S1 **ZyFISH Protocol.**
(DOC)

Video S1 **Obtaining lymphocyte nuclei for FISH.**
(MOV)

Table S1 **Troubleshooting guide.**
(DOC)

Table S2 **Lines tested with zyFISH.** *Laboratory internal nomenclature for unpublished transgenic lines. § N: Mice tested with zyFISH C: Number of mice tested with zyFISH (N) whose zygosity result matched that obtained from progeny testing or was known based on parental genotype; in three lines zyFISH results were confirmed via prospective breeding with non-transgenic mates as described in Figure 2A and Table 2; tga20: 11 mice; NGPI155: 4 mice; NGPI177: 5 mice. Not applicable (N/A).
(DOC)
