# Supplementary figures and images for: ZyFISH: A Simple, Rapid and Reliable Zygosity Assay for Transgenic Mice
Source: PLoS One. 2012 May 29;7(5):e37881. doi: 10.1371/journal.pone.0037881 (PMC3362593; doi:10.1371/journal.pone.0037881)

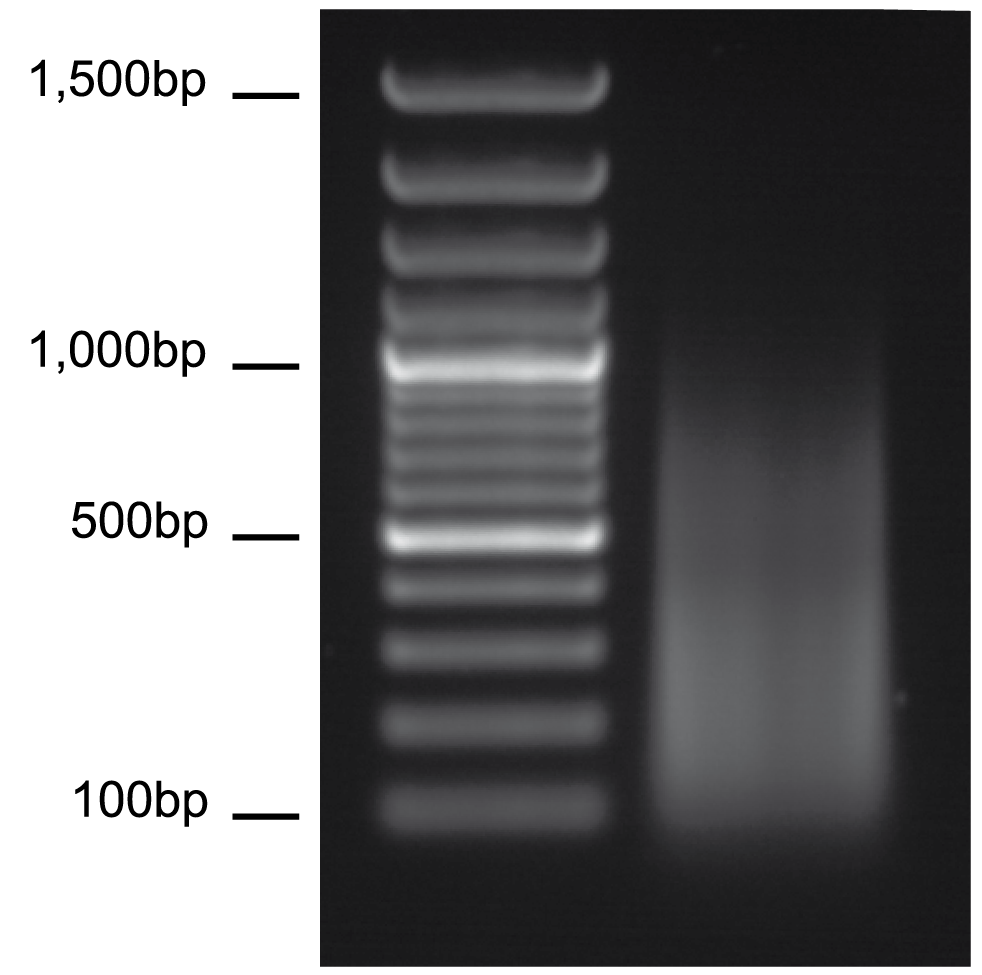

Supplement: Figure S1 — Optimal FISH probe length. A typical example of a biotin-labeled probe that produced clear signals and low background fluorescence when hybridized to fixed lymphocyte nuclei obtained from C57BL/6-tga20tg/0 mice. Following nick translation, 3 µl of probe was mixed with loading dye and denatured for 3 min at 95°C, placed on ice shortly, and then loaded alongside a 100 bp marker (100 bp plus gene ruler, Fermentas) on a 1% (w/v) agarose gel with 0.2 µg/ml ethidium bromide in TAE. Correct probe smears for FISH range from 100–1000 bp, the majority of the probe running below 500 bp. (TIF) [file pone.0037881.s001.tif]

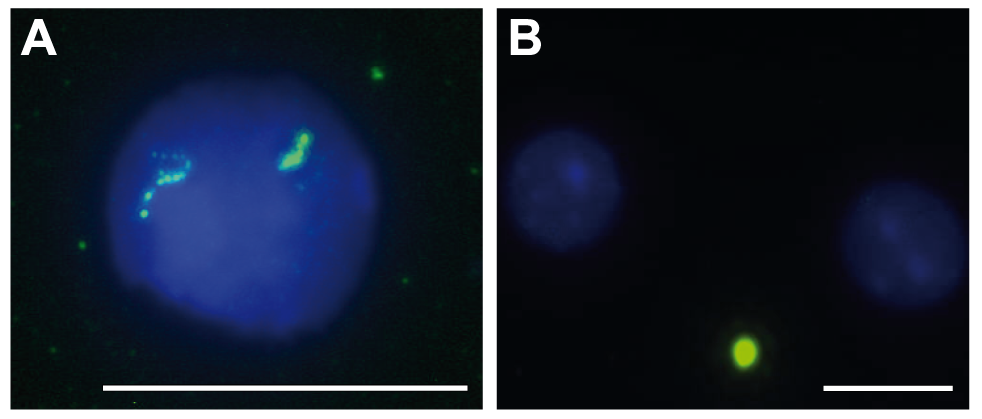

Supplement: Figure S2 — Artifacts that may influence zyFISH scoring. (A) Merged FISH image of a fixed lymphocyte nucleus from a homozygous C4 transgenic mouse carrying 25 transgene copies and a vector length of 12 kb per haploid genome [22] hybridized with a fluorescein-labeled probe. Occasionally, the hybridization signals appear as a string of hybridization spots (left signal). More often, signals will have an elongated appearance (right signal). Scale bar = 20 µm. (B) Merged FISH image with fluorescent debris. These two nuclei would be excluded from the scoring process as the debris overpowers any specific signals from within the nuclei. Scale bar = 20 µm. (TIF) [file pone.0037881.s002.tif]

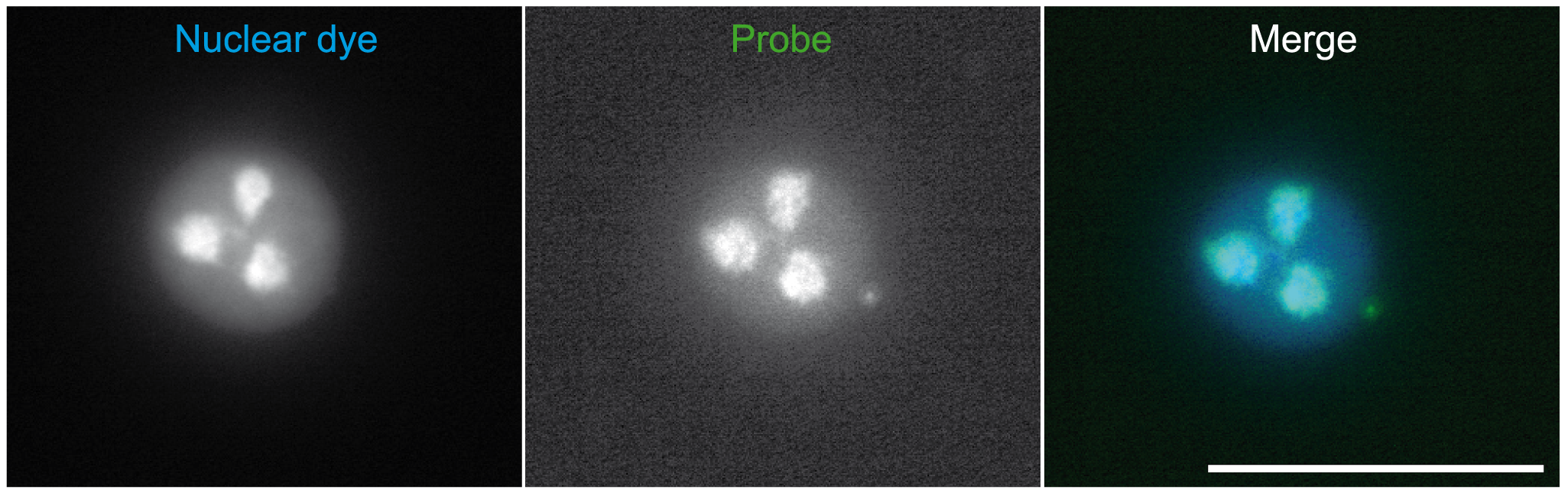

Supplement: Figure S3 — Example of mouse major satellite FISH images. FISH was performed as described in the Materials and Methods section on fixed lymphocyte nuclei from a C57BL/6 mouse with a fluorescein-labeled probe (Probe) for mouse major satellite (MMS) sequences [15] and counterstained with Hoechst-33342 (Nuclear dye), scale bar = 20 µm. The nuclear dye signal and the signal obtained with the MMS probe co-localize (Merge) within the same regions of the nucleus. The MMS probe can thus be used as a positive control for the FISH procedure when first using this assay. (TIF) [file pone.0037881.s003.tif]
