# Supplementary material for: ZyFISH: A Simple, Rapid and Reliable Zygosity Assay for Transgenic Mice
Source: PLoS One. 2012 May 29;7(5):e37881. doi: 10.1371/journal.pone.0037881 (PMC3362593; doi:10.1371/journal.pone.0037881)
Supplement: Text S1 — ZyFISH Protocol. (DOC) [file pone.0037881.s004.doc]

# ZyFISH protocol

## Reagents

- EDTA (e.g. Calbiochem, cat. no. 324503)
- Potassium chloride (e.g. Merck, cat. no. 1.04936.0500)
- Methanol (100% (v/v)) Note: Flammable, volatile and toxic.
- Acetic acid (100% (v/v); Merck Art. Nr.1.000063.1000) Note: Corrosive and flammable.
- Salmon sperm DNA (Invitrogen, cat. no. 15632011)
- Formamide (100% (v/v); Sigma-Aldrich, cat. no. 47671-1l-F) Note: Corrosive and hazardous if inhaled or ingested.
- 20× SSC Buffer (3.0 M sodium chloride, 0.30 M sodium citrate; Ambion, cat. no. AM9765)
- Ethanol absolute (100% (v/v); e.g. Analar, cat. no. 101077Y) Note: Flammable.
- Nuclease-free bovine serum albumin (BSA; Sigma, cat. no. A7030-50G)
- Dextran sulfate (sodium salt; Sigma, cat. no. D6001)
- Fluorescence mounting medium (e.g. Dako, cat. no. S3023)
- Nuclear dye (Hoechst-33342, Invitrogen, cat. no. H 1399) Note: Hoechst dyes are potentially mutagenic and carcinogenic. They must be handled with care and disposed of appropriately.
- Triton X -100 (Roche, cat. no. 789 704)
- Amberlite MB-6113 (Merck AG, cat. no. 115165)
- Nick translation mix for *in situ* probes (either Roche Applied Science, Cat. No. 11 745 808 910 for fluorescently labeled probes, or Roche Applied Science, cat. no. 11 745 824 910 for biotinylated probes)
- Fluorescent dNTP labeling mixture (e.g. containing flourescein-dUTP; Roche Applied Science, cat. no. 11 636 154 910 ) for fluorescently labeled probes
- Streptavidin Alexa Fluor 488 conjugate (Invitrogen, cat. no. S11223) for the detection of biotinylated probes.
- DNA Loading Dye (e.g. Fermentas, cat. no. R0611)
- DNA purification kit (e.g. GE Healthcare, cat. no. 28-9034-70)
- Ethidium bromide solution (e.g. Sigma, cat. no. E1510) Note: Toxic and potentially carcinogenic.
- Agarose (e.g. Invitrogen, cat. no. 15510-027)
- 100 bp DNA Ladder (e.g. GeneRuler Plus; Fermentas, cat. no. SM0324)
- Template DNA, plasmid containing the transgenic construct used for microinjection in a low salt buffer or sterile water Note: Consult your local biosafety officer for appropriate disposal of reagents containing recombinant DNA and/or plasmid DNA containing antibiotic resistance genes. Furthermore, all experiments using wild-type or transgenic mice must conform to national laws and local animal-welfare regulations.

## Equipment

- Heating block (e.g. Eppendorf, Thermomixer Compact)
- Filters (pore size: 0.45 µm, e.g. Schleicher&schuell, cat. no. 10462100)
- Surgical blades (e.g. Swann Morton Ltd., cat. no. 0207)
- Microscope slides (Superfrost Plus microscope slides; Menzel-Glaeser, cat. no. J1810AMNZ)
- Microscope slide jars and inserts
- Oven or incubator (large enough to contain the dark moist chamber at 37°C)
- Water bath (adjustable to 40°C and 70°C)
- A heat lamp within your animal facility
- Dark moist chamber
- 2-D shaker to agitate slide jars
- Spectrophotometer (e.g. Thermo Fisher Scientific, NanoDrop ND-100)
- Microcentrifuge (e.g. Eppendorf, 5415R)
- Clinical centrifuge (e.g.Thermo Electron Corporation, Multifuge 3 S-R)
- Vacuum centrifuge (e.g. Eppendorf, Concentrator 5301)
- Fluorescent microscope (e.g. Olympus BX61 TRF equipped with a CCD camera (Olympus, F-View Soft Imaging system) and a cold light source (EXFO_XI120PC-xl supplied by Olympus) )
- Image analysis software (e.g. Analysis 5.0, Olympus Soft Imaging Solutions GmbH)
- Statistical software program (e.g. Prism 5.01, GraphPad Software Inc.)

## Reagent and Equipment Setup

**Deionized formamide**

Add 10 mg of Amberlite MB-6113 beads per milliliter of 100% (v/v) formamide and agitate at RT for 30 – 40 min. Filter the suspension and store appropriate aliquots at -20°C (e.g., 45 ml or 140 ml) until use.

**Denaturation solution**

For 20 slides, fill slide jar with 200 ml deionized formamide 70% (v/v) in 2× SSC and titrate to pH 7.0 with 10N HCl. Denaturation solution can be stored at 4°C for 2 weeks and may be reused once[1].

**Blocking solution**

Prepare fresh blocking solution on the day of use containing 1% (w/v) BSA in 4× SSC.

**Hoechst-33342 stock solution**

Prepare Hoechst-33342 1% (w/v) in ddH_2_O and store in small aliquots of 10µl at -20°C. Shield from light until use.

**Hybridization solution**

Autoclaved 50% (w/v) dextran sulfate can be stored in an air tight container at RT and can be used to make fresh hybridization solution when needed. Prepare a 1% (w/v) nuclease-free BSA solution in ddH_2_O on the day of hybridization solution preparation. Prepare 5 ml of hybridization solution containing 4× SSC, 0.2% (w/v) nuclease-free BSA, 20% (w/v)) dextran sulfate at a time. Store at 4°C for up to one month[1].

**Fixative solution**

The fixative solution consists of 25% (v/v) acetic acid and 75% (v/v) methanol. Make fixative solution fresh on the day of lymphocyte nuclei preparation before the bleeding procedure and place at -20°C until cold.

**Dark moist chamber** Place multiple layers of paper towels or blotting paper on the floor of the chamber and add water without exceeding the level of paper. Place a slide rack inside and wrap the chamber in aluminum foil to shield from light.

## Procedure

### Probe Construction

1. Probe creation for fluorochrome-labeled probes (A) or for biotin-labeled probes (B). Note: Be sure to follow the appropriate detection method (A or B) for your probe later in the protocol (Step 41).

**A** Enzymatic fluorescent dye labeling with the Nick translation kit for *in situ* probes (Roche Applied science, cat. no. 11 745 808 910) according to the manufacturer’s instructions with some published [2] and our own modifications.

1. Add 5-8 µg of template DNA in a low salt buffer (e.g. TE buffer) to 3 µl of dNTP mixture (including flourescein-dUTP, e.g. Roche Applied Science, cat. no. 11 636 154 910).
2. Add sterile ddH_2_O to a volume of 80 µl and
3. Add 20 µl of nick translation enzyme mixture (Roche Applied science, cat. no. 11 745 808 910).

**B** Enzymatic biotin labeling with Biotin-Nick translation Mix for *in situ* probes (Roche Applied Science, cat. no. 11 745 824 910) according to the manufacturer’s instructions with some published [2] and our own modifications.

1. Pipette 5-8 µg of template DNA in a low salt buffer (e.g. TE buffer) into a 1.5 ml tube.
2. Add ddH_2_O to a final volume of 80 µl.
3. Add 20 µl Biotin-Nick translation mix.
4. Mix the reaction mixture carefully and incubate at 15°C for 90 min.
5. Place on ice and test the length of the probe by extracting 3 µl of the reaction mix: Add loading dye, denature at 95°C for 3 min, place on ice for 3 min spin down and analyze the size on a 1% (w/v) agarose gel with 0.2 µg/ml ethidium bromide. The optimal probe size will be a smear from 100 to 1,000bp; the majority should be lower than 500 bp (Figure S1).
6. Incubate for an additional 15 min if the appropriate probe size has not yet been achieved. Test again according to Step 3.
7. When the desired smear has been achieved, stop the reaction by adding 2 µl 0.5 M EDTA (pH 8.0) per 100 µl or by purifying the labeled probe directly with the GE Illustra purification kit according to the recommendations of the manufacturer. Elute in 50 µl of Buffer 4. DNA purification must be performed in either case.
8. Determine concentration and calculate total amount of probe by UV-spectrometry.
9. Calculate the number of slides that can be produced with your total amount of probe. For large target sequences (transgenics with more than 100 kb of transgenic DNA per haploid genome, e.g. plasmid-based transgenics with 10-12 copies per haploid genome and a construct length of 10 kb) use 100 ng / slide, for smaller target sequences use at least 200 ng / slide.
10. Mix the total amount of purified probe with 20 μg of unlabeled salmon sperm DNA.
11. Precipitate probe with cold 100% (v/v) ethanol (2-3 volumes) for 1 h at -80°C or overnight at −20°C.
12. Spin down at 16,000g for 20 min at 4°C.
13. Discard the supernatant with a pipette and dry the pellet using a vacuum centrifuge.
14. Resuspend the pellet in 10 μl 100% (v/v) deionized formamide per slide (number of slides estimated at step 7) until it is completely dissolved.
15. Heat probe-formamide mix for 5 min at 72°C to denature DNA then place on ice.
16. Add the same volume of hybridization solution as deionized formamide (see step 12) to the denatured probe. Mix well, spin down, and store at - 20°C until use. Note: Labeled probes may be stored in hybridization solution at -20°C for at least 3 months.

### Slide preparation

1. Prepare fresh fixative and place at -20 °C. Calculate 3-4 ml per mouse.
2. Blood sampling for distant (A) or proximal (B) animal facilities. See supplementary video material (Video S1), for a step by step explanation of a blood collection method and the slide preparation. The depicted bleeding procedure is fast, technically undemanding and minimally invasive, but any “blood cell-sparing” method may be used. Since only a small blood sample is required, the blood sampling method that inflicts minimal harm to the animals should be selected.

#### A. For animal facility > 1 h away from laboratory

1. Prepare one 1.5 ml tube containing 10 μl of 0.5 M EDTA, pH 8.0 per mouse to be screened.
2. Warm the mouse briefly using a heat lamp and obtain 5 drops (~100 μl) of blood from the lateral tail vein by making a small incision with a sterile scalpel. Drop the blood directly into the pre-labeled microcentrifuge tube. Invert the tube immediately or tap lightly to avoid coagulation of the sample. Blood samples may be transiently stored at 4°C before transportation. Note: Always take a sample from a confirmed hemizygous individual as a control.
3. Transport the samples cooled (2-8°C) to your lab for further processing within 24 h.
4. Upon arrival, bring each blood-EDTA-mix to ~10 ml of 65 mM KCl in a 15 ml tube and hold at room temperature (RT) for 60 min.

#### B. For animal facility < 1 h away from laboratory (adapted in part from [3])

1. Prepare one 15 ml tube containing 10 ml 65 mM KCl per mouse.
2. Warm the mouse briefly using a heat lamp and obtain 2-5 drops (~30-100 μl) of blood from the lateral tail vein by making a small incision with a sterile scalpel. Drop the blood directly into the pre-labeled 15 ml tube Note: Always take a sample from a confirmed hemizygous individual as a control.
3. Hold the suspension at RT for 60 min.
4. Centrifuge at 400 × g for 5 min at 4°C in a clinical centrifuge, discard all but 500 μl of the supernatant without disturbing the pellet.
5. Prepare 1.5 ml tubes with the appropriate labeling. Place your tubes on a rack on ice, place cold fixative on ice.
6. Gently resuspend cells in the residual solution by flicking the tube. Slowly add 500 μl of ice-cold, fresh fixative, flicking the tube intermittently to prevent clumping.
7. Transfer suspension carefully into a 1.5 ml tube.
8. Centrifuge for 2 min at 1500 × g at 4°C
9. Gently remove most of the supernatant with a P1000 pipette, being careful not to disturb the pellet. Gently resuspend in the residual (100 μl) fixative by flicking the tube.
10. Add 100 μl of cold fixative and resuspend, then add 1 ml of fixative and invert tube.
11. Centrifuge for 2 min at 1500 × g, 4°C.
12. Gently remove most of the supernatant with a P1000 pipette, being careful not to disturb the pellet. Gently resuspend in the residual fixative by flicking the tube.
13. Add 1 ml cold fixative and invert tube.
14. Centrifuge for 2 min at 1500 × g, 4°C.
15. Remove all but 50-100 μl of fixative, and gently resuspend the nuclei in the remainder. Note: Lymphocyte nuclei suspension can be stored up to at least 11 months at -20°C without interfering with the assay (see expected results). If you wish to store nuclei, add an additional 1 ml of fixative to the nuclei suspension. Repeat steps 24 – 28 after storage before preparing new slides (step 29).
16. Drop ~50 µl of nuclei suspension (3-4 drops) on clean microscope slides (Superfrost Plus, Menzel-Glaeser), label with pencil. Superfrost slides are pre-cleaned and do not require emersion or cleaning.
17. Let slides air dry at RT in a closed slide box. This step is performed overnight. Continue with the hybridization in the afternoon of the following day.

### Hybridization and detection

The hybridization and detection procedure is performed essentially as previously described [1] with some minor changes.

1. Place 70% (v/v) ethanol at -20°C until cold.
2. Heat an oven or an incubator to 37°C and place dark moist chamber inside.
3. Prepare denaturation solution and warm to 70°C in a water bath.
4. Prepare dehydration sequence for slides (Jars containing: 70% ice cold ethanol can be placed in the ice box; followed by 80%, 95%, 100% (v/v) ethanol at RT).
5. Place slides with interphase lymphocyte nuclei for 2 min in denaturation solution prewarmed to 70°C. Note: This step is critical. Low denaturation temperatures can result in poor hybridization efficiency. High denaturation temperatures can lead to poor nuclei morphology and low nuclei density on slides.
6. Place slides for 2 min in ice-cold 70% (v/v) ethanol (step 31).
7. Dehydrate slides by incubating for 2 min each in RT 80%, 95%, and 100% (v/v) ethanol.
8. Allow slides to dry in a covered (but not sealed) slide box until ethanol is completely evaporated.
9. Add 20 μl of probe mix to the center of each slide. Avoid making air bubbles. Keep remaining probe mix on ice and place an appropriately cut piece of parafilm on the probe mix and allow probe to distribute across the slide by capillary force. Any air bubbles can be removed with gentle pressure. Place slides face-up in the dark moist chamber and incubate overnight at 37°C. Continue with step 41 within 14-18h.
10. Heat two slide jars containing 250 ml of 2× SSC and 50% (v/v) formamide (non-deionized), 2× SSC to 39°C in a water bath.
11. Remove slides from moist chamber and proceed with protocol variants (A) for fluorescently labeled probes or with (B) for biotin-labeled probes.

#### A. Fluorescently labeled probes

1. Remove parafilm, place in slide jar inserts and wash slides at 39°C in 50% (v/v) formamide and then in 2× SSC for 15 min each.
2. Continue wash for 15 min in 39°C 2× SSC and then for 15 min at RT in 1× SSC.
3. Allow slide to equilibrate in 4× SSC at RT for at least 5 min.

#### B. Biotin-labeled probes

1. Prepare blocking solution: 1% (w/v) BSA in 4× SSC. To dissolve BSA, place the solution in the water bath at 39°C and vortex periodically.
2. Remove parafilm and incubate slides at 39°C in 50% (v/v) formamide, 2× SSC for 15 min.
3. Incubate slides for 15 min at 39°C in 2× SSC followed by 15 min at RT in 1× SSC.
4. Allow slides to equilibrate for at least 5 min at RT in 4× SSC.
5. Cover slide with warm blocking solution (about 2 ml per slide) and incubate for 30 min in the dark moist chamber at 37°C, OR incubate in a slide jar containing blocking solution for 30 min at 37°C.
6. Dilute Alexafluor 488-conjugated streptavidin (SA-488) 1:200 in blocking solution (50 μl per slide).
7. Remove slides one by one from the dark chamber and dispose of excess blocking solution by knocking the side of the slides on a paper towel. Add 50 μl diluted SA-488 to each slide and cover with parafilm. Incubate for 1 h in the dark moist chamber at 37°C.
8. Agitate slides in 0.1% (v/v) Triton X-100, 4× SSC in a light protected jar on a 2D shaker to wash away unbound SA-488 for 10-15 min at RT and then twice in 4× SSC for 10-15 min at RT.
9. Counter-stain chromosomal DNA with Hoechst-33342 50 ng/ml in 4× SSC at RT for 5-10 min in a light protected slide jar (e.g. aluminum foil wrapped).
10. Rinse in 1× SSC to remove excess Hoechst-33342. Blot slides by knocking sideways on a paper towel.
11. Add 1-2 drops of fluorescent mounting medium (Dako) to stained slide, and apply a cover slip, avoiding bubbles. Squeeze excess mounting media out by pressing gently on cover slip. Note: Slides are ready to be examined after the cover slip has been mounted and the mounting medium has hardened, but it may be stored protected from light at 4°C for at least 3 weeks for scoring at a later time point.

### Scoring and Statistical Evaluation

1. Examine slides using a fluorescent microscope by alternating between the Blue/Cyan filter (identification of eligible nuclei) and the green filter (scoring of nuclei).
2. Apply the following rules for the scoring of lymphocyte nuclei:

- In order to be eligible for scoring, nuclei must be intact and should neither overlap nor touch other nuclei, as visualized by nuclear dye in the Blue/Cyan filter.
- Once you have identified eligible nuclei within your field of view switch to the green filter for scoring.
- Hybridization signals are required to be uniformly bright among different nuclei. In case of multiple signals in the same nucleus, these should also display equal intensity. Remember to vary the focal plane during nuclei evaluation, as hybridization signals within one nucleus may not necessarily be aligned at a single point on the Z-axes. Keep in mind that transgenic loci with multiple tandem insertions may be seen as a “string of beads” or have an elongated appearance [3,4]. This could wrongly lead to the multiple scoring of the same signal specific for a single locus (Figure S2A).
- Nuclei with auto-fluorescent debris in the vicinity should be excluded, as this can obscure hybridization signals (Figure S2B).
- After reaching your target number of nuclei, score the remaining nuclei within your field of view to avoid a sampling bias.

1. Nuclei from each slide are then categorized into one of two groups depending on the number of signals they display. Nuclei determined to have two hybridization signals will make up the group of positively-scoring nuclei (POS). These are the only nuclei that indicate that an animal is homozygous. Nuclei containing 0, 1, 3 or more signals will constitute the group of negatively-scoring (NEG), as they do not indicate homozygosity. See Figure 1 for examples of nuclei and how to score them.
2. Compare the two groups (POS and NEG) from each slide to the two groups from the hemizygous control slide using a two-tailed Fisher’s exact test by entering your data into a contingency table. This test assesses the significance of association between the two groups (POS and NEG) from each slide and will provide you with an exact p-value or < 0.0001 if the value is smaller than 0.0001. Your significance level α should be = 0.001.
3. Homozygous animals are defined as animals that are significantly different from a known hemizygous control animal as calculated with Fisher’s exact test. The remaining animals will be hemizygous by default, if they were found to be positive for the transgene by conventional PCR genotyping.

## Time considerations

The time requirements for a complete zyFISH assay are shown in supplementary Table 1. The assay was designed to be performed over the course of 3 days and allows the handling of up to 40 samples by a skilled investigator.

## Troubleshooting

Most problems will become apparent toward the end of the assay (Step 45). Troubleshooting advice can be found in supplementary Table S1.

# References

1. Knoll JH, Lichter P (2005) In situ hybridization to metaphase chromosomes and interphase nuclei. Curr Protoc Hum Genet Chapter 4: Unit 4 3.

2. Cremer M, Grasser F, Lanctot C, Muller S, Neusser M, et al. (2008) Multicolor 3D fluorescence in situ hybridization for imaging interphase chromosomes. Methods Mol Biol 463: 205-239.

3. Paris D, Toyama K, Megarbane A, Casanova PM, Sinet PM, et al. (1996) Rapid fluorescence in situ hybridization on interphasic nuclei to discriminate between homozygous and heterozygous transgenic mice. Transgenic Res 5: 397-403.

4. Swiger RR, Tucker JD, Heddle JA (1995) Detection of transgenic animals without cell culture using fluorescence in situ hybridization. Biotechniques 18: 952-954, 956, 958.
