# Supplementary material for: ZyFISH: A Simple, Rapid and Reliable Zygosity Assay for Transgenic Mice
Source: PLoS One. 2012 May 29;7(5):e37881. doi: 10.1371/journal.pone.0037881 (PMC3362593; doi:10.1371/journal.pone.0037881)
Supplement: Table S1 — Troubleshooting guide. (DOC) [file pone.0037881.s006.doc]

## Table S1. Troubleshooting guide.

| **Problem** | **Possible reasons / Solutions** |
| --- | --- |
| Poor nuclei morphology | Check nuclei morphology on an additional slide before denaturation to identify if the problem is pre- or post-denaturation. |
|  | Pre-denaturation: Optimize bleeding procedure and ensure that your blood samples do not coagulate. Make fresh 65 mM KCl solution and fresh fixative from clean reagents. |
|  | Post-denaturation: Optimize denaturation temperature. If the denaturation temperature of 70°C is exceeded, it may result in loss of nuclei density on the slide and poor nuclear morphology. With a high denaturation temperature, some nuclei will have a hollow appearance. Make sure coverslip is applied only with gentle pressure. |
| No hybridization signal | Check identity of DNA template for nick translation by restriction enzyme fragment analysis or sequencing. |
|  | Check probe length and concentration (steps 3 and 6). This can only be performed before the addition of salmon sperm DNA. |
|  | Make sure probe is completely resuspended in deionized formamide at step 12. |
|  | Increase the amount of probe per slide. |
|  | Check that denaturation temperature is sufficiently high. |
|  | Validate hybridization protocol and reagents with known probe and target sequence (e.g. MMS probe). |
|  | In the case of small target sequences, consider detecting with an amplification technique (e.g. biotin-conjugated anti-fluorescein antibody and SA-488; this may be performed in an analogous manner to the biotin detection steps detailed in this protocol). |
| High background on slide, positive for the nuclear dye | Include an additional wash / fixation cycle for nuclei before dropping them onto the slides, or take a smaller blood sample. |
|  | Ensure that the hypotonic KCl treatment is long enough. |
| High background on slide, positive for the probe spectrum | Check that the majority of the probe length is shorter than 500 bp. |
|  | Make sure the slides have not dried out during hybridization. |
|  | Increase stringency and temperature of post-hybridization washes. |
|  | For biotin-labeled probes detected with avidin-fluorochrome conjugates: Increase stringency and duration of post-detection washes. In this case, a certain homogenous background can be expected and tolerated if the single intranuclear signals in nuclei from a hemizygous control mouse are clear and bright. |
