# Supplementary material for: ZyFISH: A Simple, Rapid and Reliable Zygosity Assay for Transgenic Mice
Source: PLoS One. 2012 May 29;7(5):e37881. doi: 10.1371/journal.pone.0037881 (PMC3362593; doi:10.1371/journal.pone.0037881)
Supplement: Table S2 — Lines tested with zyFISH. *Laboratory internal nomenclature for unpublished transgenic lines. § N: Mice tested with zyFISH C: Number of mice tested with zyFISH (N) whose zygosity result matched that obtained from progeny testing or was known based on parental genotype; in three lines zyFISH results were confirmed via prospective breeding with non-transgenic mates as described in Figure 2A and Table 2; tga20: 11 mice; NGPI155: 4 mice; NGPI177: 5 mice. Not applicable (N/A). (DOC) [file pone.0037881.s007.doc]

## Table S2. Lines tested with zyFISH

| **Name of Line** | **Transgenic copy number per haploid genome** | **Samples tested with zyFISH** | **Can be assessed with zyFISH** | **Number of zyFISH-tested mice with confirmed zygosity status (N/C) §** | **Reference for the transgenic line** |
| --- | --- | --- | --- | --- | --- |
| *tg*a20 | 30 | 80 | Yes | 28/28 | Fischer M. et. al. EMBO J, 1996 [2] |
| C4 | 25 | 3 | Yes | 3/3 | Flechsig E. & Schmerling D. et al. Neuron, 2000 [22] |
| NGPI155* | 18 | 83 | Yes | 18/18 | Unpublished line |
| NGPI177* | 12 | 56 | Yes | 24/24 | Unpublished line |
| H-LTαβ L19* | unknown | 33 | Yes | 6/6 | Unpublished line |
| Tg40 | 6 | 2 | Yes | 2/2 | Baumann F. et al., PLoS ONE, 2009 [17] |
| Tg42 | 5 | 2 | Yes | 2/2 | Baumann F. et al., PLoS ONE, 2009 [17] |
| Tg1047 | 3 | 3 | No | N/A | Baumann F. et al., EMBO J, 2007 [18] |
| AlbLTαβ | 2 | 20 | No | N/A | Heikenwälder M. & Zeller N. et al., Science, 2005 [21] |
